# Unlocking the Potential Role of Decellularized Biological Scaffolds as a 3D Radiobiological Model for Low- and High-LET Irradiation

**DOI:** 10.3390/cancers16142582

**Published:** 2024-07-18

**Authors:** Alexandra Charalampopoulou, Amelia Barcellini, Andrea Peloso, Alessandro Vanoli, Stefania Cesari, Antonia Icaro Cornaglia, Margarita Bistika, Stefania Croce, Lorenzo Cobianchi, Giovanni Battista Ivaldi, Laura Deborah Locati, Giuseppe Magro, Paola Tabarelli de Fatis, Marco Giuseppe Pullia, Ester Orlandi, Angelica Facoetti

**Affiliations:** 1 CNAO National Center for Oncological Hadrontherapy, Radiobiology Unit, Research and Development Department, 27100 Pavia, Italy; angelica.facoetti@cnao.it; 2 Hadron Academy PhD Course, School for Advanced Studies (IUSS), 27100 Pavia, Italy; 3 Department of Internal Medicine and Therapeutics, University of Pavia, 27100 Pavia, Italy; lauradeborah.locati@unipv.it; 4 CNAO National Center for Oncological Hadrontherapy, Radiation Oncology Unit, Clinical Department, 27100 Pavia, Italy; ester.orlandi@cnao.it; 5 Division of Visceral Surgery, Department of Surgery, Geneva University Hospitals, 1205 Geneva, Switzerland; andreapeloso@hotmail.it; 6 Unit of Anatomic Pathology, Department of Molecular Medicine, University of Pavia, 27100 Pavia, Italy; alessandro.vanoli@unipv.it (A.V.); s.cesari@smatteo.pv.it (S.C.); 7 Unit of Anatomic Pathology, Fondazione IRCCS Policlinico San Matteo, 27100 Pavia, Italy; 8 Unit of Histology and Embryology, Department of Public Health, Experimental and Forensic Medicine, University of Pavia, 27100 Pavia, Italy; antonia.icaro@unipv.it; 9 Department of Biology and Biotechnology “L. Spallanzani”, University of Pavia, 27100 Pavia, Italy; margarita.bistika01@universitadipavia.it; 10 Cell Factory, Fondazione IRCCS Policlinico San Matteo, 27100 Pavia, Italy; stefania_croce186@yahoo.it; 11 Department of General Surgery, Fondazione IRCCS Policlinico San Matteo, 27100 Pavia, Italy; lorenzo.cobianchi@unipv.it; 12 Department of Clinical, Surgical, Diagnostic and Pediatric Sciences, University of Pavia, 27100 Pavia, Italy; 13 Collegium Medicum, University of Social Sciences, 90-419 Łodz, Poland; 14 Istituti Clinici Scientific Maugeri IRCCS, Radiation Oncology Department, 27100 Pavia, Italy; giovannibattista.ivaldi@icsmaugeri.it; 15 Medical Oncology Unit, Istituti Clinici Scientific Maugeri IRCCS, 27100 Pavia, Italy; 16 CNAO National Center for Oncological Hadrontherapy, Medical Physics Unit, Clinical Department, 27100 Pavia, Italy; giuseppe.magro@cnao.it; 17 Medical Physic Unit, Istituti Clinici Scientific Maugeri IRCCS, 27100 Pavia, Italy; paola.tabarelli@icsmaugeri.it; 18 Research and Development Department, CNAO National Center for Oncological Hadrontherapy, 27100 Pavia, Italy; pullia@cnao.it

**Keywords:** 3D model, bioscaffolds, high LET, low LET

## Abstract

**Simple Summary:**

Two-dimensional (2D) models are unable to mimic the intricacies of the reaction in the natural tumor microenvironment to the irradiation, biological and architectural complexity, or dynamic nature of the many tissues. These features can be recreated using a decellularized extracellular matrix (ECM), such as bioscaffolds. We hypothesized that bioscaffolds can be a feasible and effective three-dimensional (3D) model, worthwhile also for radiobiological aims. To test our hypothesis, two cell lines (HMV-II and PANC-1) were seeded in decellularized porcine liver-derived scaffolds and irradiated with carbon ions (high-LET irradiation) and photons (low-LET irradiation). For the first time in the literature, we found that the 3D environment provided by the bioscaffolds was suitable for radiobiological research as well as being cost-effective. This model provides the opportunity to explore the biological consequences of different radiation modalities over prolonged periods of time.

**Abstract:**

Introduction: Decellularized extracellular matrix (ECM) bioscaffolds have emerged as a promising three-dimensional (3D) model, but so far there are no data concerning their use in radiobiological studies. Material and Methods: We seeded two well-known radioresistant cell lines (HMV-II and PANC-1) in decellularized porcine liver-derived scaffolds and irradiated them with both high- (Carbon Ions) and low- (Photons) Linear Energy Transfer (LET) radiation in order to test whether a natural 3D-bioscaffold might be a useful tool for radiobiological research and to achieve an evaluation that could be as near as possible to what happens in vivo. Results: Biological scaffolds provided a favorable 3D environment for cell proliferation and expansion. Cells did not show signs of dedifferentiation and retained their distinct phenotype coherently with their anatomopathological and clinical behaviors. The radiobiological response to high LET was higher for HMV-II and PANC-1 compared to the low LET. In particular, Carbon Ions reduced the melanogenesis in HMV-II and induced more cytopathic effects and the substantial cell deterioration of both cell lines compared to photons. Conclusions: In addition to offering a suitable 3D model for radiobiological research and an appropriate setting for preclinical oncological analysis, we can attest that bioscaffolds seemed cost-effective due to their ease of use, low maintenance requirements, and lack of complex technology

## 1. Introduction

In vitro experience in understanding the biological effects of radiotherapy (RT) is mainly based on two-dimensional (2D) cell culture experiments. Indeed, 2D models are the most widely approaches used in radiobiology for their easy handling, relatively low cost, and because they are supported by numerous standardized protocols and assays. However, they are not able to replicate the biological and architectural complexity, the dynamic nature of the different tissues, and the complexities of the response in the native tumor microenvironment (TME) to the irradiation. In recent years, efforts have been made to fully replicate a physiologically relevant and high-throughput in vitro system for the study of cell responses and interactions after and during oncological treatments [1]. In this context, spheroids and organoids are becoming increasingly attractive as three-dimensional (3D) radiobiological models [2,3,4], but, to the best of our knowledge, there are no data on the use of decellularized extracellular matrix (ECM) bioscaffolds. 

The ECM is a network of proteins, glycoproteins, and proteoglycans organized in a 3D ultra-structure that provides structural and functional cell support and also provides a reservoir for angiogenic and growth factors that are critical for cell differentiation [5,6]. Additionally, the ECM is involved in cell-to-matrix cross-talk promoting cell proliferation, migration, and differentiation and impacting inflammation and immune modulation [7]. Thus, there is a strong dynamic reciprocity between cells and the ECM [8]. For these reasons, in both preclinical and clinical studies, several different tissue types have been successfully reconstructed using xenogeneic and allogeneic ECMs, and 3D decellularized bioscaffolds becoming increasingly attractive for regenerative medicine and tissue or organ bio-engineering [9,10].

Given these properties, ECM bioscaffolds seem to be an ideal model for radiobiological preclinical studies. To test whether a natural 3D-bioscaffold might be an effective tool for radiobiological analysis and might achieve an assessment that could be as close as possible to what occurs in vivo, we seeded two well-known radioresistant cell lines in decellularized porcine liver-derived scaffolds and irradiated them with both high- and low-Linear Energy Transfer (LET) radiation. The LET of ionizing radiation indicates how much energy (per unit/length) is deposited through which it passes [11]. Compared to low LET, the lower dependence on fractionation and cell-cycle stage, as well as the decreased oxygen enhancement factor (OER), made high LET attractive for the treatment of cancers that are generally resistant to photons [12]. Among the radioresistant histologies, vaginal malignant mucosal melanoma and pancreatic adenocarcinoma were chosen for our testing, having in common rarity, poor prognosis, and better response, as reported in the literature, with carbon ion RT (CIRT) than low LET [13,14].

## 2. Materials and Methods

### 2.1. Biological Scaffolds

The Foundation I.R.C.C.C Policlinico San Matteo of Pavia (Italy) provided biological scaffolds as a 3D in vitro model. These scaffolds derive from the decellularization of the porcine liver, a procedure that generates an ECM-based, acellular, 3D unit preserving unaltered natural organ-specific architecture with regard to bioactive macro- and micromolecules, as well as hierarchical anatomical architecture, partitioned into multiple subunits [6]. The scaffolds were initially maintained in penicillin/streptomycin antibiotic solution and conditioned for 24 h before cell seeding cells by overnight incubation in a fresh complete growth medium.

### 2.2. Cells and Reagents

The human malignant melanoma cell line (HMV-II) was purchased from Sigma-Aldrich (St. Louis, MO, USA), and the human pancreatic adenocarcinoma cell line (PANC-1) from the Experimental Zooprofilactic Institute of Lombardy and Emilia Romagna. Both cell types were grown in a humidified environment at 37 °C and 5% CO_2_ with RPMI 1640 medium the first, and DMEM the second, containing heat-inactivated fetal bovine serum (FBS), 100 U/mL penicillin, and 0.1 mg/mL streptomycin. Culture media and all supplements were obtained from Sigma Aldrich. Cells were split at confluence using 10% trypsin. Before irradiation, cells were tested for mycoplasmas using 300 nM of fluorescent 4′,6-diamidino-2-phenylindole (DAPI) and found negative.

### 2.3. Cell Seeding

After the overnight incubation, scaffolds were removed from the conditioning growth medium and each of them was placed into a well of a 48-well culture plate (Corning, Milan, Italy). 10^6^ cells were suspended in 50 μL–100 μL of fresh growth medium and seeded onto each scaffold. Scaffolds were then incubated at 37 °C to allow cell attachment, and 2 h later complete growth medium was added to keep scaffolds completely immersed. The medium was changed every 2–3 days. 

### 2.4. Irradiations

Scaffolds were irradiated with X-rays (low LET) and carbon ions (high LET) beams. X-ray irradiation (X-RT) was performed with a 6 MV linear accelerator (3 Gy/min as dose rate) at the Radiotherapy Department of the Istituti Clinici Scientifici Maugeri of Pavia (Italy). Flasks were horizontally positioned on a 1.5 cm thick layer of plexiglass to ensure the electronic equilibrium at Source Flask Distance (SFD) of 101.5 cm with the beams irradiating from below (180°). CIRT was performed at the National Center for Oncological Hadrontherapy (CNAO) of Pavia (Italy) using the fixed horizontal clinical beamline with the active scanning technique. Flasks were positioned in a water phantom with its entrance window located at the room isocenter. To produce a homogeneous irradiation zone that was 6 cm thick, a Spread-Out Bragg peak (SOBP) was formed by changing 31 beam energies (246–312 MeV/n, or 120–180 mm) in increments of 2 mm water-equivalent path lengths. Scaffolds were placed at a depth of 15 cm corresponding to the mid of the SOBP. This configuration provided a complete liquid/plastic interface with no ion deflection due to the presence of air. Positioning the flask at the center of the SOBP minimized dose gradients, ensuring uniform exposure, which is essential for accurate assessment, necessarily focusing on a single LET value rather than varying LETs. 

**Figure 1:**
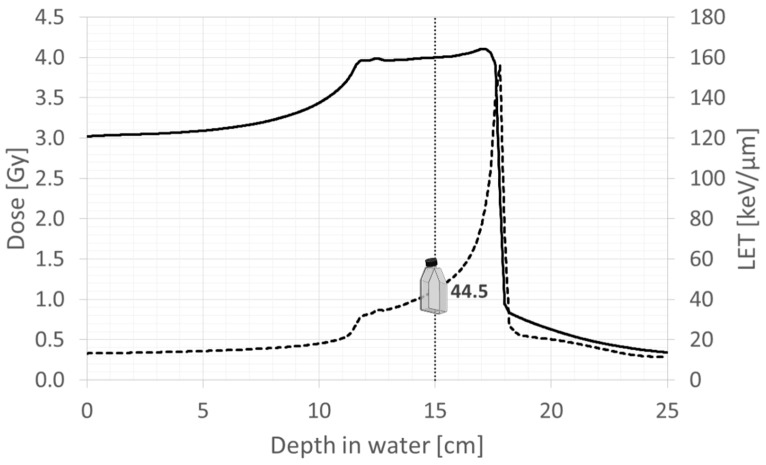
Irradiation of the bioscaffolds with X-ray (low LET) and carbon ion (high LET) beams. Low LET has been quantified as 0.2 keV/μm and high LET as 44.5 keV/μm.

Before irradiation, scaffolds were placed in flasks (Corning, Milan, Italy), and the medium was added to completely cover them when placed either in a vertical (in the case of CIRT), or horizontal position (for X-RT). Scaffolds were exposed to 2 Gy and 4 Gy for both radiation types for both low- (X-RT) and high-LET (CIRT), which were quantified as 0.2 keV/μm [15] and 44.5 keV/μm, respectively (RayStation V11B, RaySearch Laboratories, Stockholm, Sweden) (Figure 1). Within the scope of our study, the LET of 44.5 keV/μm provided a clinically pertinent condition, supporting our goal to effectively differentiate between low and high LET. The selected SOBP extensions reflected clinically relevant irradiation beams, ensuring that our experimental conditions applied to therapeutic settings. 

For each cell line and each experimental condition, data were obtained by three independent experiments and each experiment was run in triplicate.

### 2.5. Post-Irradiation

Directly after either type of irradiation, the FBS-free medium was aspirated and the scaffolds were placed in the 48-well culture plate, incubated with fresh growth medium, and preserved in the incubator. Control samples were observed by confocal microscope 5 weeks after seeding to assess cell distribution. Hoechst 33342; Sigma Aldrich, Milan, Italy was used for staining, and images were acquired with the Leica TCS SP8 DLS microscope at the Centro Grand Strumenti of the University of Pavia, and the 3D structure was constructed. Both control and irradiated samples were fixed with 4% formalin every 7 days for 4 weeks (3 repeats for each condition). After fixation, scaffolds were stored in the refrigerator at 4 °C until processing. The biological scaffolds were then embedded in paraffin and full-thickness tissue sections were performed to obtain serial 4–5 μm slides. To observe and compare the impact of the two types of RT on the different cellular structures, sections of the biological scaffolds were stained with hematoxylin and eosin (H&E), periodic acid-Schiff (PAS), Masson’s trichrome, Alcian blue, and Picrosirius red (PSR). Melanin absorbance was measured at 450 nm using media samples collected from the scaffold 7 days after exposure to both types of RT and filtered. Reading was automatically performed by BioTek Gen5 Data Analysis Software, version 3.0. 

### 2.6. Statistical Analysis

For the aim of the current work, only descriptive analysis, based on the evaluation of the confocal analysis and the histological specimens, was performed. At least 3 slices per condition and staining were evaluated. Two expert pathologists revised independently the histological specimens.

## 3. Results

### 3.1. Cell Repopulation within the Scaffolds

**Figure 2:**
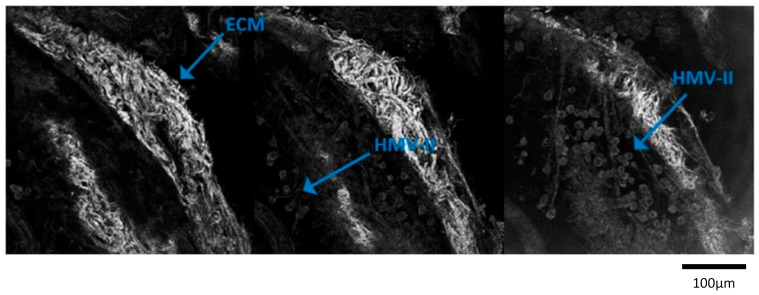
Images at different depths of scaffolds seeded with HMV-II cells were obtained with confocal microscopy to evaluate whether and how cancer cells populated the scaffold. The figure showed that cells were able to intercalate deeper in the structure of the bioscaffolds from the surface through the decellularized extracellular matrix (ECM). This image was captured at three different successive planes. Scale bar: 100 µm.

We observed how HMV-II and PANC-1 were able to repopulate the decellularized scaffolds, integrating the structures at full thickness and remaining viable up to the 5 weeks of observation (Figure 2). Sham-irradiated seeded scaffolds (0 Gy condition) did not undergo any alteration of the morphological structures and both the analyzed cell lines maintained different morphological features consistent with the analyzed cytotypes, without dedifferentiation. Indeed, regarding the bioscaffold structure, we documented the preservation of the ECM micro-architecture and the presence of collagen on hepatic ECM; we also confirmed the collagen structural preservation (H&E), the absence of linear collagen fibrils in the parenchymal space by Masson’s trichrome and PSR staining, and the persistence of acid mucins by Alcian blue staining.

Moreover, histology indicated that the cells can recreate the expected neoplastic architectures with coherent staining within the bioscaffolds.

**Figure 3:**
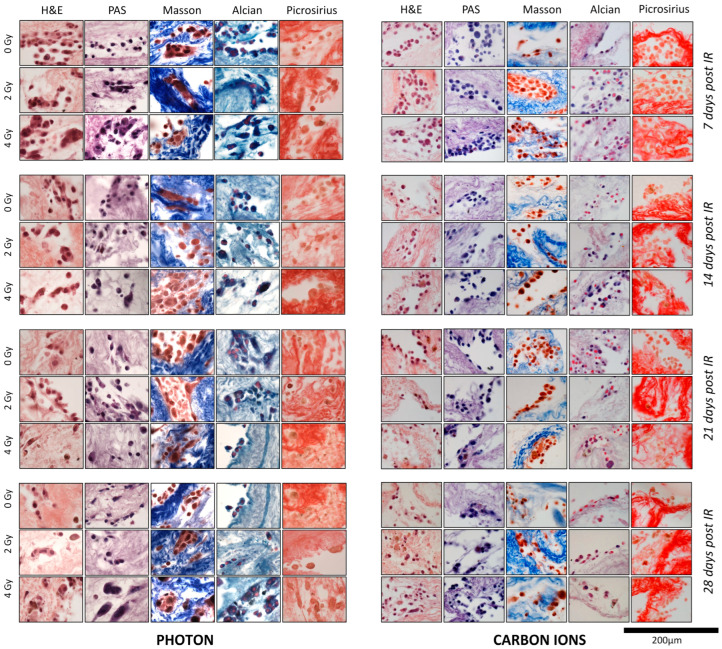
Histological sections of scaffolds seeded with HMV-II cells, after photon (**left**) and carbon ion (**right**) irradiation (IR) at each time point. To evaluate the effects of C-ion irradiation vs. photon irradiation histological sections of populated scaffolds at 7, 14, 21, and 28 days were stained with hematoxylin and eosin (H&E), periodic acid-Schiff (PAS), Masson’s trichrome, Alcian blue, and picrosirius red (PSR). Refer to Table 1 for the specific description of the histological characteristics of each section.

**Figure 4:**
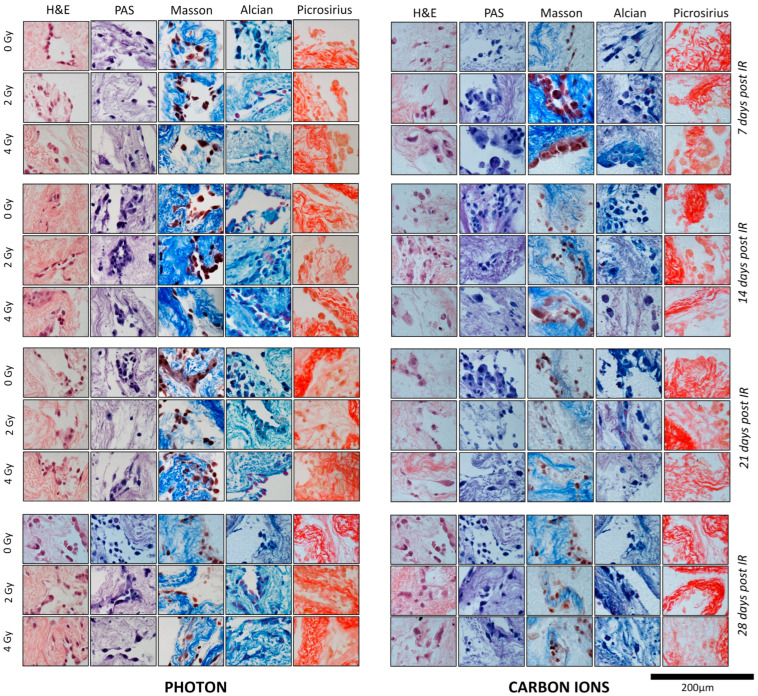
Histological sections of scaffolds seeded with PANC-1 cells, after photon (**left**) and carbon ion irradiation (**right**) at each time point. To evaluate the effects of different irradiation (IR) conditions, the histological sections of populated scaffolds at 7, 14, 21, and 28 days were stained with hematoxylin and eosin (H&E), periodic acid-Schiff (PAS), Masson’s trichrome, Alcian blue, and picrosirius red (PSR). At 28 days, the experiments were conducted in parallel; thus, the same control condition (0 Gy) was applied. Refer to Table 2 for the specific description of the histological characteristics of each section.

In particular:

(i) In HMV II scaffolds stained with H&E, we observed the cellular and tissue pattern coherent with the foreseen histology. We assessed an infiltrative attitude of the cells within the scaffolds that were large and epithelioid, with prominent nucleoli and abundant eosinophilic cytoplasm, the latter characterized by a granular feature (expression of melanin in the melanosomes that are about to fall apart). These aspects increased over the time of observation (Figure 3).
(ii) In PANC-1-scaffold sections, H&E staining documented the organization of the cells into ducts, which showed evident PAS+ and Alcian-blue, and an infiltrative behavior by PSR. Over time, tumor cells increased in number and size and displayed aggressive features (Figure 4).

### 3.2. Analysis after Low and High LET Irradiation

#### 3.2.1. HMV-II Cell Line

No significant morphological changes were observed in the images of the stained sections after 2 Gy exposure to low- and high-LET irradiation. The first morphological alterations were assessed from 14 days after 4 Gy in both conditions with different features according to the LET used, as summarized in Table 1. Specifically, the damage after 4 Gy of X-RT was milder and delayed, with cytoplasm desegregation (after 14 days), foamy (for the presence of numerous vacuoles and lipid droplets) cytoplasm (after 21 days), the presence of polynucleated cells, and cells with three-times greater physiological size, as well as cytoplasm dissociation (after 28 days). On the other hand, after 14 days of 4 Gy CIRT, the alterations were more serious and characterized by increased nucleus sizes, binucleations, diapedesis, and cytoplasm desegregation. Moreover, when compared with X-RT, a more significant foamy cytoplasm was recorded at 21 days and substantially altered nuclei, binucleations, and higher cytoplasm desegregation after 28 days. Additionally, melanin synthesis occurred earlier in the X-RT group (14 days against 21 days, respectively) (Figure 3).

#### 3.2.2. PANC-1 Cell Line

Consistent with previous 2D experiences [16,17,18], CIRT had a more severe effect on PANC-1 cells than X-RT in terms of cellular alteration. Indeed, no significant post-actinic damage was observed during the X-RT irradiations at any time point or dose considered. However, earlier and more massive cytological effects were recorded in scaffolds subjected to CIRT.

As summarized in Table 2, we found after 2 Gy of CIRT: atypical nuclei in cells were characterized by an increased volume (14 days), a reduction in total cell number (21 days), and clear cell deterioration (28 days). The post-actinic changes increased at the increasing dose (4 Gy) and at the increasing time after exposures, with atypical and hyperchromatic nuclei, multinucleation, and bigger cell volume at 7 days; more atypical cells with triple nucleus size at 14 days; a greater reduction in cell number at 21 days; and a pronounced cell deterioration at 28 days (Figure 4).

## 4. Discussion

In this study, we assessed the feasibility of using a decellularized porcine liver-derived scaffold as a 3-D radiobiological model to evaluate the effects of low- and high-LET irradiations on radioresistant cancer cell lines. Firstly, our study first demonstrated that biological scaffolds provide a favorable 3D environment for cell proliferation and expansion. A major concern regarding the application of bioscaffolds in biological research is whether the intensive decellularization might induce biomolecule denaturation or compromise the ECM microarchitecture, thereby impeding appropriate cell growth or leading to cell differentiation back into the scaffold tissue of origin [6]. Nevertheless, morphological assessment by two independent expert pathologists confirmed the structural integrity of the liver scaffolds as evidenced by hematoxylin and eosin, Masson’s trichrome, picrosirius red, and Alcian blue staining. These findings are consistent with the current literature [19]. Moreover, cells did not show signs of dedifferentiation and retained their distinct phenotype. In the control condition, we appreciated the infiltrative behaviour of the HMV-II and PANC-1 cells within the scaffolds with the capability to propose a histological normal architecture and the increased aggressiveness as time progressed coherently with their anatomopathological and clinical aspects (Table 1 and Table 2; Figure 3 and Figure 4). Compared to the 2D radiobiological models, 3D bioscaffolds provide an ECM that plays a dual role in supporting cells, both structurally and biochemically. The ECM facilitates cellular communication, cell–matrix adhesion, and the synthesis of new ECM components [20,21]. It also provides a modulating environment for cellular migration, proliferation, and differentiation [6,22], in part due to the intrinsic presence of matrix-bound growth factors [23]. Having established the viability of the bioscaffolds as a conducive environment milieu for cellular growth and proliferation, we proceeded with exposure to low- and high-LET radiations to assess the possibility of implementing these 3D models in radiobiological studies. CIRT has a higher relative biological effectiveness (RBE), being able to produce clustered DNA damage that is difficult to repair, compared to simple DNA double-strand breaks (DSBs) of X-RT- For these reasons, CIRT is potentially advantageous for radioresistant histologies (i.e., tumors with low α/β ratio [12] and a high rates of hypoxic cells [24]). Mucosal malignant melanomas and pancreatic adenocarcinomas, characterized by intrinsic radioresistance, represented compelling subjects to test the different responses to low and high LET in this new 3D model. The HMV-II and PANC-1 cellular responses to X-RT and CIRT provided promising data to support our hypothesis. 

In HMV-II cells, the histological examination of the sections after irradiation with 2 Gy of both X-RT and CIRT showed no significant morphological differences compared to the control group. However, actinic changes became more pronounced with escalating doses of low and high LET and with increased exposure time. In particular, CIRT induced severe cytological damage including extensive cytoplasm disaggregation, diapedesis, and increase in nuclei size, as well as the appearance of a relevant number of binucleated cells (Table 1). Furthermore, melanin production started earlier in the X-RT cohort, with pigmentation detectable already at 14 days (vs. the 21 days in the CIRT group). These observations, the muted response at low doses and the accelerated melanin synthesis after X-RT, are consistent with the biological and clinical behaviour of vaginal mucosal melanomas, which are known to be highly radioresistant and aggressive, often presenting as variably pigmented lesions [14,25]. High-dose CIRT has been proven to be more effective than X-RT in providing long-term local control of mucosal malignant melanoma [26,27,28]. Radiobiologically, 2D in vitro studies have shown that CIRT reduced cellular viability, proliferation, and migration in a dose-dependent manner [29]. Melanin, particularly eumelanin, is radioprotective and photoprotective due to its potent antioxidant properties and function as a natural sunscreen. Melanogenesis has been shown to inhibit apoptosis, promote cellular proliferation [30,31] and induce hypoxia [32]. In our experiments, X-RT was identified as an early activator of melanogenesis, in contrast to CIRT, suggesting that the early production of melanin after X-RT and the delayed, reduced melanogenesis after CIRT contribute to the mechanism of radioresistance to X-RT and the radiosensitivity to CIRT of this challenging malignancy. 

Concerning the response of PANC-1 cells to CIRT, it was observed that the cytopathic effects induced were more severe than those induced by X-RT and manifested earlier compared to HMV-II, as shown in Table 2. While after 2 Gy of X-RT, the first damage was visible after 28 days, after the same dose of CIRT, the situation dramatically worsened at 24 days showing atypia and changes in the cell volume up to a substantial cell deterioration of the surviving fractions at the following time points. Moreover, after 4 Gy of CIRT, the tumor cells within the scaffolds drastically deteriorated: we recorded atypia, hyperchromatism of the nuclei, multinucleation, and a significant reduction in the number of the cells that were irretrievably damaged. The increased sensitivity of pancreatic cells to CIRT is consistent with previous observations. The radioresistance of pancreatic adenocarcinoma was proved in several preclinical and clinical scenarios [13,18,24,29,33,34]. This intrinsic hallmark has been attributed to a variety of factors, including alteration of DNA damage response mechanisms, DNA repair processes, and cell-cycle checkpoints, as well as hypoxia and the induction of stellate cells that result in fibrosis [35]. However, in several pancreatic cell lines, CIRT has been reported to reduce cancer cell viability, proliferation, and migration compared to photon-based therapy [17,18,29]. Clinically, CIRT has demonstrated favourable outcomes in both neoadjuvant settings [34,36] and in a radical one [37] and has achieved encouraging results compared to X-RT alone. This suggests that CIRT represents a compelling treatment alternative for patients with malignancies that are recalcitrant to conventional therapies. 

Besides proving the feasibility of the porcine liver scaffolds as a fertile environment for preclinical oncological analysis and an appropriate 3D model for radiobiological study, we can confirm that bioscaffolds appeared to be cost-effective, being simple to handle, and requiring little maintenance and no sophisticated technology. Unlike 2D models, the 3D models used in this study more closely mimic the in vivo environment. Bioscaffolds promoted cell viability and proliferation, while also allowing the study of tumor cell infiltration patterns within ECM (Figure 2). The cells recapitulated the in vivo tumor architecture—a phenomenon not detectable by any 2D preclinical model—and the scaffolds provided a suitable environment for oxygen, nutrients, and cell growth factor diffusion, thus permitting an evaluation of the post-irradiation effects over an extended period of time (Figure 3 and Figure 4). 

## 5. Conclusions

For the first time, this study provides that porcine liver scaffolds serve as a 3D model suitable for radiobiological research. This model provides the opportunity to explore the biological consequences of different radiation modalities over prolonged periods. Given the promising results of our study and the intriguing properties of the biological bioscaffolds, the future direction may include co-culturing different cell lines to better mimic a tumor microenvironment (TME) that closely resembles the clinical scenario.

## Figures and Tables

**Table 1:** Morphological HMV-II alteration after XRT and CIRT.

	0 Gy	2 Gy		4 Gy	
	Control Condition	Photons Irradiation	Carbon Ions Irradiation	Photons Irradiation	Carbon Ions Irradiation
7 days	The cells infiltrated the scaffolds	No significant variation compared to the control	No significant variation compared to the control	No significant variation compared to the control	No significant variation compared to the control
14 days	Granular cytoplasm and larger nuclei (indicating the expression of melanin in the imploding melanosomes).	No significant variation compared to the control	No significant variation compared to the control	Cytoplasm desegregation coupled with melanin release.	Increased nucleus size, cytoplasm desegregation, and binucleations.
21 days	Increased the previous evidence	No significant variation compared to the control	No significant variation compared to the control	Morphological cell’s shape changes (epithelia-morphic), foamy cytoplasm	Foamy cytoplasm, starting melanin production
28 days	Increased the previous evidence	No significant variation compared to the control	No significant variation compared to the control	Polynucleated cells, tripled size, cytoplasm dissociation, higher melanin production.	Altered nuclei, significant binucleations. Higher cytoplasm desegregation and increased melanin production.

**Table 2:** Morphological PANC-1 alterations after XRT and CIRT.

	0 Gy	2 Gy		4 Gy	
	Control Condition	Photons Irradiation	Carbon Ions Irradiation	Photons Irradiation	Carbon Ions Irradiation
7 days	Pancreatic cells infiltrate the scaffolds	No significant variation compared to the control	No significant variation compared to the control	No significant variation compared to the control	Atypical and hyperchromatic nuclei, cell volume increase, multinucleation.
14 days	Pancreatic cells arrange themselves into the typical architecture of neoplastic tissue.	No significant variation compared to the control	Atypical nuclei, overall cell volume increase.	No significant variation compared to the control	More atypical cells, triplicated nucleus size
21 days	There are ducts visible, and as the cancerous cells become larger and more aggressive	No significant variation compared to the control	Reduction in cell number	No significant variation compared to the control	Significant reduction in cell number
28 days	Increased the previous evidence	More atypical cells, no actinic damage	Clear cell deterioration	More atypical cells, no actinic damage	Pronounced cell deterioration

## Data Availability

Data will be shared upon reasonable request made to the corresponding author.
